# Reproductive risks in 35-year-old adults born very preterm and/or with very low birth weight: an observational study

**DOI:** 10.1007/s00431-020-03864-5

**Published:** 2020-11-07

**Authors:** Sylvia M. van der Pal, Sanne A. van der Meulen, Sophie M. Welters, Leonhard A. Bakker, Christianne J. M. de Groot, Anton H. van Kaam, Erik (G.H.W.) Verrips

**Affiliations:** 1 TNO Child Health, PO Box 3005, NL, 2301 DA Leiden, The Netherlands; 2 Department of Obstetrics and Gynecology, Amsterdam UMC, Vrije Universiteit Amsterdam, Amsterdam Reproduction & Development Research Institute, Amsterdam, The Netherlands; 3 Department of Neonatology, Emma Children’s Hospital, Amsterdam UMC, University of Amsterdam, Amsterdam, The Netherlands

**Keywords:** Reproduction, Very preterm, Very low birth weight, Preterm-born adults

## Abstract

Evidence suggests that increased survival over the last decades of very preterm (VPT; gestational age < 32 weeks)– and very low birth weight (VLBW; birth weight < 1500 g)–born infants is not matched by improved outcomes. The objective of our study was to evaluate the reproductive rate, fertility, and pregnancy complications in 35-year-old VPT/VLBW subjects. All Dutch VPT/VLBW infants born alive in 1983 and surviving until age 35 (*n* = 955) were eligible for a POPS-35 study. A total of 370 (39%) subjects completed a survey on reproductive rate, fertility problems, pregnancy complications, and perinatal outcomes of their offspring. We tested differences in these parameters between the VPT/VLBW subjects and their peers from Dutch national registries. POPS-35 participants had less children than their peers in the CBS registry. They reported more problems in conception and pregnancy complications, including a three times increased risk of hypertension during pregnancy.

*Conclusion*: Reproduction is more problematic in 35-year olds born VPT/VLBW than in the general population, possibly mediated by an increased risk for hypertension, but their offspring have no elevated risk for preterm birth.

**Table:** 

**What is known:** *At age 28, the Dutch national POPS cohort, born very preterm or with a very low birth in 1983, had lower reproductive rates than the general Dutch population (female 23% versus 32% and male 7% versus 22%)*. **What is new:** *At age 35, the Dutch POPS cohort still had fewer children than the general Dutch population (female 56% versus 74% and male 40% versus 56%). Females in the POPS cohort had a higher risk of fertility problems and pregnancy complications than their peers in the Dutch national registries, but their offspring had no elevated risk for preterm birth.*

**What is known:**

*At age 28, the Dutch national POPS cohort, born very preterm or with a very low birth in 1983, had lower reproductive rates than the general Dutch population (female 23% versus 32% and male 7% versus 22%)*.

**What is new:**

*At age 35, the Dutch POPS cohort still had fewer children than the general Dutch population (female 56% versus 74% and male 40% versus 56%). Females in the POPS cohort had a higher risk of fertility problems and pregnancy complications than their peers in the Dutch national registries, but their offspring had no elevated risk for preterm birth.*

## Introduction

Preterm birth is the major cause of neonatal deaths in the Western world. Over a third of the estimated neonatal mortality is associated with preterm birth complications, such as respiratory distress syndrome, intraventricular hemorrhages, necrotizing enterocolitis, and sepsis [1]. The estimated rate of preterm births in Europe is 8.7% (690.931 cases) and over 11% in North America in 2014. From the total number of preterm deliveries in Europe, 16% was very preterm (VPT), i.e., gestational age (GA) < 32 weeks [2].

Advanced perinatal technology and improved neonatal care greatly increased the survival rate of VPT and VLBW infants over the last decades. Current evidence suggests that increased survival is not always matched by an improved outcome [3–7]. Therefore, long-term follow-up studies after preterm birth are relevant sources of information on physical health, well-being, and health-related quality of life of VPT/VLBW infants who are now adults [8]. Some of the less favorable outcomes do not show until adulthood, for instance, reduced reproductive rates. Only a few studies [9, 10] assessed reproductive outcomes of VPT/VLBW adults [11] and these studies are often based on large registry cohorts [12–14] without detailed information on possible complications that influence reproductive rates. Long-term cohort studies on fertility and pregnancy of adults born VPT and/or VLBW are scarce.

In the Netherlands, consequences of VPT/VLBW birth have been extensively studied in the Project on Preterm and Small for Gestational Age (POPS) which comprises a nationwide multicenter birth cohort, born in 1983 [15]. The POPS cohort included 94% of all live-born VPT/VLBW infants in the Netherlands that year. Several POPS publications documented problems in various domains, such as pulmonary morbidity and neurodevelopmental outcomes at several ages until adulthood [15, 16]. One study on reproductive outcome in POPS females at a relatively young age of 28 years found lower reproductive rates than in the general population [17]. In an effort to further contribute to the evidence on reproductive rates in adults born VPT/VLBW, the objective of the present study was to evaluate differences in reproductive rate, fertility, and pregnancy complications between 35-year-old VPT/VLBW POPS subjects and their peers from the Dutch National Population Registry (CBS) [18] and Dutch Perinatal PeriNed Registries [19].

## Methods

### Subjects

The population consisted of all VPT and/or VLBW infants born alive in the Netherlands in 1983 [15]. The Project on Preterm and Small for Gestational Age Infants (POPS) study achieved to include 1338 infants (94%) of this population. The remaining 85 (6%) infants could not be included due to administrative problems. The mortality rate was similar in both groups (26%) and the differences in mean values of gestational age and birth weight did not reach statistical significance at the alpha < 0.05 level between the two groups [20]. In the year of their turning 35 years of age, all 955 eligible male and female POPS participants were invited to participate in the POPS-35 study. An invitation was sent out to the last known e-mail or postal addresses. After 1 month, additional attempts were made to reach the non-responding participants by phone or text message. If a phone number was not available, participants were approached through social media, mainly LinkedIn. As a result of these procedures, 370 subjects (39% of 955 eligible) agreed to participate in the study. After signing the informed consent form, participants received a link to access an online questionnaire. The questionnaire was accessible from November 2018 until May 2019. Female participants completed a slightly different questionnaire than males, because of phrasing differences, for instance, “your pregnancy” versus “your partner’s pregnancy.”

We compared the reproduction rates of our POPS-35 sample to those available from the Dutch National Population Registry (CBS) [18]. Total numbers on being married and the mean number of children of 35-year-old Dutch females/males in 2018 were sent by CBS on request. The age of the first child in the general population in 2018 was obtained from CBS’s open databank Statline [18].

The incidence of pregnancy and delivery complications in POPS-women regarding their first live-born child was compared with data from the Dutch National Perinatal Registration PeriNed [19]. PeriNed is a Dutch national register of birth care for which maternal and neonatal data are collected by obstetricians and pediatricians in the Netherlands. Total numbers, means, and standard deviations for nulliparous (P0) women < 35 years of age who gave birth after ≥ 22 weeks of gestational age in 2013 were sent by PeriNed on request. The year 2013 was chosen because of digital changes in registration after 2013 that affected the registration. PeriNed warns for underreporting with regard to rare pregnancy complications and outcomes for the mother [19].

It was not possible to isolate POPS participants from the registry data, due to strict privacy protection regulations. The Medical Ethics Committee of the Leiden University Medical Center had approved previous POPS study protocols and the Medical Ethics committee of TNO approved the subsequent amendment concerning the POPS-35 study.

### Questionnaire

Self-reported reproductive outcomes included in the online questionnaire were as follows: relationship (yes or no, and living together and/or married), infertility (none, unsuccessfully tried to conceive for over 1 year), conception (unknown, spontaneous, assisted), pregnancy complications (ever had a miscarriage, ever had a stillbirth, hypertension, (pre)-eclampsia, gestational diabetes), delivery mode (unknown, vaginal, instrumental, caesarian), placental pathology (yes, no), number of children (number, age at first childbirth), and offspring perinatal outcome (gestational age, birth weight, NICU admission).

The choice for these outcome measures was based on data available from national population registries and from a questionnaire study about pre-eclampsia [21]. Gestational hypertension and gestational diabetes were selected as pregnancy complications as these are reported in the Dutch Perinatal Registration PeriNed. Furthermore, previous POPS studies showed increased risks for hypertension and diabetes [22, 23] and also outcome measures were derived from the context of the Developmental Origins of Health and Adult Disease (DOHAD) hypothesis that links risks for disease later in life with environmental conditions in early life [24].

### Statistical analysis

POPS participants are generally very willing to contribute to follow-up assessments, but in cohort studies, attrition always occurs. Fortunately, the POPS cohort is well documented and potential selection characteristics were available from several assessments in the past. Thus, differences between participants (*n* = 370) and eligible non-participants (*n* = 585) in distributions of gestational age, birth weight, sex, disability (derived from the HUI questionnaire at age 14), and parental level of education (at POPS’ age 14) could be evaluated. Differences between participants and non-participants in the distributions of continuous variables were tested by means of independent samples two-sided *t* tests for equal variances, categorical variables by Pearson’s chi-squared tests, and ordered categorical variables by linear-by-linear association tests. Differences between the POPS cohort and the data of their peers from the CBS and PeriNed registries were tested with one-sample two-sided *t* tests for continuous variables and Pearson’s chi-squared tests for categorical variables. A difference was considered statistically significant if the *p* value was below 0.05.

All analyses were performed using SPSS version 25.

## Results

**Fig. 1:**
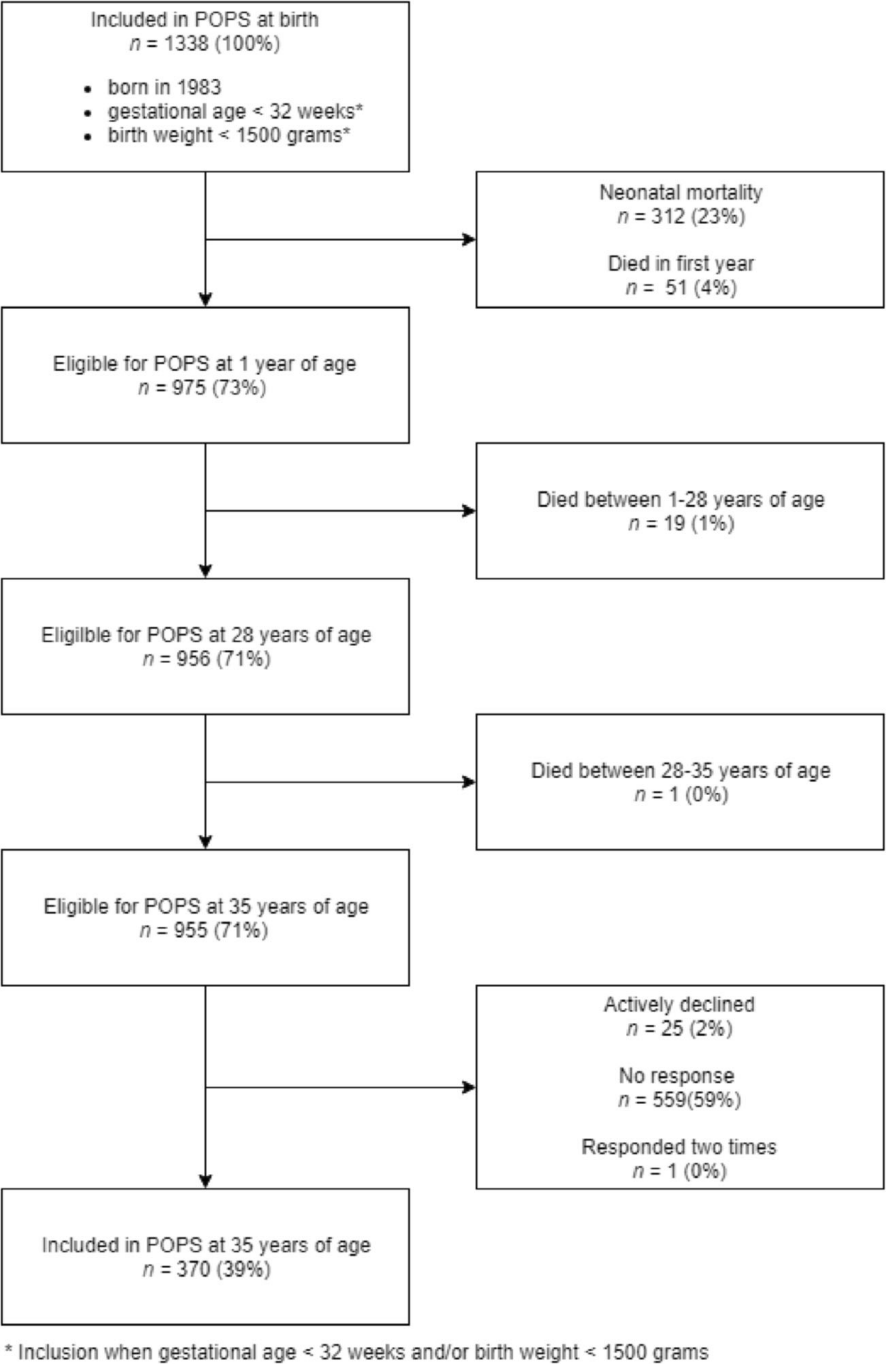
Sampling frame of the POPS-35 study

Figure 1 shows the eligibility of subjects and participation rates.

A total of 370 subjects (39% of 955 eligible) agreed to participate in the study. The characteristics of the participants in the POPS-35 study and eligible non-participants are presented in Table 1.

**Table 1:** POPS-35 participants versus non-participants

	POPS-35 participants *N* = 370	Non-participants *N* = 585	*p* value
Gestational age, *m* (sd)	31.05 (2.4)	31.04 (2.6)	0.95
Birth weight, grams, *m* (sd)	1325 (297)	1306 (274)	0.32
Sex, *n* (%)			< 0.001
Female	209 (56%)	251 (43%)	
Male	161 (44%)	334 (57%)	
Disability at 14 yrs, *n* (%)^^^			0.003*
None	129 (37%)	161 (33%)	
1–2 small disabilities	135 (38%)	165 (33%)	
> 2 small or 1 severe	68 (19%)	108 (22%)	
> 1 severe disability	19 (5%)	59 (12%)	
Parent educational level 14 years, *n* (%)^†^			< 0.001*
Low	89 (25%)	251 (49%)	
Middle	148 (41%)	169 (33%)	
High	125 (35%)	90 (18%)	

^^^Disabilities on 8 HUI attributes at 14 years; no participation at 14 years: participants *n* = 19, non-participants *n* = 92

^†^Low: primary education, technical and vocational training, lower and middle general secondary education; Middle: middle vocational education, higher general secondary education, pre-university education; High: higher vocational education, university; Unknown: participants *n* = 8, non-participants *n* = 75

*Chi-square linear-by-linear association

Data presented in Table 1 indicate that POPS-35 participants did not differ significantly from non-participants with regard to mean gestational age and birth weight. However, non-participants had more disabilities and were more often male. The educational level of parents was higher in POPS-35 participants than in non-participants.

Table 2 shows the reproductive outcomes of POPS-35 participants and their peers as documented by the CBS registry, stratified by sex.

**Table 2:** Number (*n*) and percentage (%) of categories of reproductive outcomes of POPS-35 females and males

Group	Characteristic	POPS-35	CBS	*p* value
Females	*N* total	*209*	*100,818*	
	Relationship, *n* (%)			
	No committed relationship	44 (21%)		
	Relationship, no cohabitation	12 (6%)		
	Relationship, with cohabitation	66 (32%)		
	Married	87 (42%)	49,978 (50%)	0.02
	Number of own children^~^, *m* (sd)	1.08 (1.1)	1.48	< 0.001
	*N* total ever tried to conceive	*141*		
	Sub-/infertility (tried > 1 year), *n* (%)	36 (26%)		
	*N* total ever pregnant	***131***		
	Ever miscarriage (< 16 weeks), *n* (%)	34 (26%)		
	Ever stillbirth (> 16 weeks), *n* (%)	0 (0%)		
	*N* total ≥ 1 child	*118 (56%)*	*74,276 (74%)*	*< 0.001*
	Age at first child, *m* (sd)	29.5 (3.3)	29.9	0.22
Males	*N* total	*161*	*101,988*	
	Relationship, *n* (%)			
	No committed relationship	49 (30%)		
	Relationship, no cohabitation	7 (4%)		
	Relationship, with cohabitation	39 (24%)		
	Married	66 (41%)	42,260 (41%)	0.91
	Number of own children^~^, *m* (sd)	0.74 (1.2)	1.04	0.002
	*N* total ever tried to conceive	*80*		
	Sub-/infertility (tried > 1 year), *n* (%)	13 (16%)		
	*N* total partner ever pregnant	*69*		
	Partner ever miscarriage (< 16 weeks), *n* (%)	18 (26%)		
	Partner ever stillbirth (> 16 weeks), *n* (%)	3* (4%)		
	*N* total ≥ 1 child	*65 (40%)*	*57,484 (56%)*	*< 0.001*
	Age at first child, *m* (sd)	30.7 (3.3)	32.7	< 0.001

*The partner of one male had experienced both miscarriage and stillbirth

~Zero if no children

Table 2 shows that the POPS-35 female group was less often married and they had fewer children than their peers in the Dutch population. The POPS-35 male group was younger when their partner had their first child and they also had fewer children than their peers in the Dutch population.

A quarter of the 141 POPS-35 females who had ever tried to conceive reported problems in getting pregnant (tried for > 1 year) and 16% of POPS-35 males reported such problems with their partner. A quarter of both POPS-35 females and partners of males went through a miscarriage in one or more pregnancies. Twenty POPS females visited a fertility clinic, nine of whom had fertility issues; in three cases, this concerned their male partners, while in eight cases, no medical cause was found. Six POPS males visited a fertility clinic, of whom two had fertility issues; in two cases, this concerned their partner and in two cases, the cause remained unknown.

In Table 3, self-reported pregnancy and delivery complications in the first pregnancy of POPS-35 and of 2013 PeriNed registry females are presented.

**Table 3:** Number (*n*) and percentage (%) of categories of pregnancy and delivery outcomes first live-born child of female POPS-35 participants

Characteristics	First pregnancy of POPS-35 women	First pregnancy of PeriNed women < 35 years	*p* value
Total *N*	118	66,561	
Conception, *n* (%)			0.002
Spontaneous	102 (86%)	49,349 (94%)	
Assisted reproductive technology	16 (14%)	3421 (6%)	
Unknown		13,791	
Pregnancy complications, *n* (%)			
Hypertension during pregnancy	28* (24%)	5980 (9%)	< 0.001
(Pre)-eclampsia^~^	17 (14%)	279 (0.4%)	< 0.001
Gestational diabetes	6 (5%)	1709 (3%)	0.08
Delivery outcome *n* (%)		66,177	
Delivery mode			0.053
Vaginal	66 (56%)	42,929 (66%)	
Instrumental	25 (21%)	10,042 (16%)	
Cesarean	27 (23%)	11,696 (18%)	
Unknown		1510	
Complications			
Placental pathology	5 (4%)	2096 (3%)	0.51
Perinatal outcomes in offspring			
Gestational age, *m* (sd)	39.3 (1.9)	38.9 (2.2)	0.04
Birth weight, *m* (sd)	3270.8 (601.4)	3304.5 (596.6)	0.54
Low birth weight (< 2500 g), *n* (%)	12 (10%)	5216 (8%)	0.36
Preterm birth (< 37 weeks), *n* (%)	9 (8%)	6016 (9%)	0.58
NICU admission of child, *n* (%)	13 (11%)	1094 (2%)	<0.001

*One woman already knew she had high blood pressure before the pregnancy

^~^In POPS-35 questionnaire: have you had pre-eclampsia/pregnancy poisoning/high blood pressure with protein in the urine/high blood pressure with convulsions (resembles an epileptic attack); (pre-)eclampsia if one of these symptoms indicated. PeriNed warns for underreportage of pregnancy complications, such as (pre-)eclampsia

Data from Table 3 indicate that of all 118 pregnancies of firstborn children of POPS-35 females, 16 pregnancies (14%) were supported by artificial reproductive technology (ART; ovarian induction, IUI, IVF, ICSI), which is twice as high as the 7% live births supported by ART/medical assistance reported in PeriNed.

In 28 pregnancies (24%), the mother experienced hypertension during pregnancy, which is higher than the 9% reported in PeriNed. Furthermore, 17 (14%) women had a pregnancy complicated by (pre-)eclampsia, compared to 0.4% in PeriNed. Six women (5%) had gestational diabetes, which is not significantly different from the 3% in PeriNed.

POPS-35 females did not differ from PeriNed females regarding delivery mode or placenta pathology during delivery. Their first child had a similar mean birth weight to those in PeriNed, but POPS-35 children were born after a longer pregnancy duration and were more often admitted to the NICU.

The firstborns of nine POPS-35 females (8%) were born preterm, namely before 37 weeks of gestation, which is comparable to 9% preterm births in PeriNed. Thirteen POPS-35 females who had children (11%) gave birth to a preterm born infant in one or more of their pregnancies.

Of the 118 females who had given birth to any child, 45 were born small for gestational age (SGA), 72 appropriate for gestational age (AGA), and for 1 female this was unknown. Neither pregnancy complications nor perinatal outcomes of their firstborn child differed between SGA and AGA women. The pregnancy of the first child of the females born SGA was not more often supported by ART (both SGA and AGA 13% ART) and a comparable percentage of females experience hypertension during pregnancy (24% for SGA and 23% for AGA), but the numbers were small. Furthermore, the first child of females born SGA did not differ from those of females born AGA with respect to mean GA at birth (39.0 weeks for SGA and 39.4 weeks for AGA, *p* = 0.36) and mean birth weight (3148 g for SGA and 3344 g for AGA, *p* = 0.09).

## Discussion

This study showed that VPT- and/or VLBW-born 35-year-old adults had a lower reproductive rate than the national population. A previous study on the same POPS cohort at age 28 already found lower reproductive rates in POPS females than in the general population [17]. The current study at 35 years of age showed that, at a group level, no substantial catch-up in reproduction had occurred over 7 years in the POPS cohort. Scandinavian registry studies also found lower reproduction rates among VPT/VLBW subjects than in the general population [12, 14].

Pregnancy and childbirth are major life events. The quality of life of women in general during pregnancy has been reported to be good to excellent [25], but pregnancy-related symptoms and anxiety are indicators of poor quality of life trajectories [26]. Given the impact of reproduction on an individual’s well-being and life course, the mechanisms behind reduced reproductive rates in VPT/VLBW subjects need exploration. Biological causes reported in the literature include disturbed sex hormone patterns or other endocrine sub-normality in female as well as male VPT/VLBW subjects [27, 28]. This is in line with the Barker or Developmental Origins of Health and Adult Disease (DOHAD) hypothesis, postulating long-term effects on health and well-being due to adaptation in growth during fetal life and infancy [24, 29]. Socio-economic factors, such as education and socioeconomic status, have also been reported to be associated with reproductive rate [30, 31]. In addition, psycho-social factors need consideration. Reduced reproduction rates at the group level may well be the result of well-informed and preference-based decisions of individual VPT/VLBW 35-year-old subjects not to have children (yet). Their parents are likely to have told them several stories about their difficult start in life and many VPT/VLBW subjects experience health problems. As a result, they may be hesitant about having a child and postpone a decision to try and get pregnant. After all, at age 35, females are still in their reproductive age. Moreover, recent evidence suggests VPT-/VLBW-born adults generally lag behind in economic achievements such as wealth [32] and also in their psychosocial development [4, 33] in comparison with their peers from the general population. One study on the POPS cohort at age 19 and 28 also showed less risk-taking and less criminal behavior as well as later engagement in romantic relationships and sexual behavior [34, 35]. The current study also shows a lower percentage of married VPT-/VLBW-born females. These outcomes might be caused by handicaps or overprotective parenting, as a result of their vulnerable start in life. Similar results were found in Finland [36].

Our study showed that, despite an increased risk of pregnancy complications, the offspring of adult VPT/VLBW females had no elevated risk of preterm birth. This is an important and positive outcome that needs wide dissemination among medical practitioners and it needs to be replicated in future research.

While postponement of reproduction may be a personal choice, fertility problems are not. Failure to conceive was previously reported to be associated with low quality of life, severe emotional distress, and irrational motherhood cognitions (i.e., “I need a child in order to lead a happy life; your world collapses when you get your period again”) [37]. We found a lower rate of spontaneous conception among VPT/VLBW females than in PeriNed and a quarter of all females indicated that they tried more than a year to get pregnant, or were still trying. It should be noted here that PeriNed warns for under-reporting of ART and that the results within the current study were self-reported. However, self-report has been shown concordant with gynecologist reports [21]. Personalized pre-conception counseling may be offered, individually by the general practitioner or in centering groups, in order to optimize the chance of getting pregnant. Smoking cessation, alcohol abstinence, reduction of caffeine, drug and medication intake, a healthy diet and weight control, reduction of psychological stress, and regular exercise are among the behaviors to be targeted [38].

We found much higher rates of gestational hypertension and (pre-)eclampsia in pregnant POPS-35 females than reported in PeriNed. It should again be noted that PeriNed warns for potential under-reporting of (early) pregnancy complications, but such under-reporting probably also occurred in POPS-35, since concordance between self-report and care professional-report has been documented [21, 39]. The prevalence of pre-eclampsia is expected to vary between 3 and 5% [40], which is still significantly lower than the percentage of (pre-)eclampsia in the pregnancy of the first child of POPS-35 females. A previous POPS study already showed increased risks for hypertension at 19 years [23] and a combined analysis of several cohorts of adults born with a VLWB also showed an increased risk for hypertension [41]. Boivin et al. described a higher risk of gestational diabetes with OR 2.34 (95% CI: 1.65–3.33) and (pre-)eclampsia with OR 1.79 (1.19–2.69) in pregnancies of VPT-born mothers [42]. In the current study, the first offspring of women who experienced (pre-)eclampsia or gestational hypertension during their first pregnancy were born with a significant lower GA and BW (data not shown). However, the net prevalence of these conditions in our study was too small to have an effect on gestational age or birth weight at the group level. Nevertheless, this should be a caveat for clinicians to carefully monitor blood pressure in the pregnancies of VPT/VLBW women, or even before their first pregnancy.

The study by Boivin also showed more pregnancy complications within females born SGA [42]. The current study found no significant differences but the numbers of pregnancy complications with females born SGA are low. The birth weight of the first child of POPS-35 females born SGA was 196 g lower but this difference was not statistically significant, possibly because of low numbers.

POPS-35 females reported higher rates of NICU admissions among POPS-35 offspring than in PeriNed, possibly because of higher rates of pregnancy complication and higher rates of instrumental and caesarian deliveries (not significant). However, the self-reported reasons for NICU admissions included neonatal problems that usually require admission to a high/medium care unit in a general hospital, and not so much a NICU, such as a late preterm birth. This might suggest that the difference between these levels of neonatal care was not so clear for some parents, which may have confounded the results.

Due to the fact that the POPS-35 participants will be in their reproductive age for at least five more years, one last follow-up is needed in order to draw final conclusions about the reproductive outcomes of the POPS cohort and to be able to identify which extra care is needed. To obtain sufficient statistical power—a well-known and inconvenient issue in studies of VPT/VLBW birth cohorts—POPS is cooperating in a European Union–funded RECAP-preterm platform (Research on European Children and Adults born Preterm; www.recap-preterm.eu) and in addition to a worldwide research collaboration on adult outcomes of VPT/VLBW birth (www.apic-preterm.org). Such collaborations may be helpful in consolidating all European and worldwide data available on VPT-/VLBW-born children and adults, in order to develop more effective, evidence-based, and personalized interventions.

### Strengths and limitations

A major strength of the study is the variety of detailed reproductive outcomes that could be measured, providing a comprehensive overview of influential factors on the reproductive outcome of preterm born adults. Another strength is the possibility to look far back in time [43], enabled by a follow-up study of the nationwide Dutch POPS cohort, that had been followed up from birth until the age of 35.

One of the limitations was the relatively small number of women who had been pregnant in our POPS-35 study and who had experienced problems. That might have nullified smaller differences in fertility or pregnancy complications. Moreover, because of this, we made no differentiation between POPS participants who were spontaneous or iatrogenic VPT born, both of which have different underlying pathophysiology, or born small for gestational age.

There were no differences in mean gestational age nor birth weight between POPS-35 participants and non-participants. Therefore, selection bias by biological variables that are hypothesized by DOHAD to be related to these parameters [24] probably did not occur and the external validity of our study was satisfactory. However, POPS-35 non-participants were more often male, had more disabilities, and are lower educated parents than the participants. This might have resulted in an underestimation of complicated fertility, pregnancy, and childbirth.

Another limitation is that no matched control group is available for the POPS cohort. Fortunately, national registries were available for comparison, which is second best to a matched control group. However, the PeriNed registry is completed by physicians, while the collected data is self-reported, both of which show under-reportage of early pregnancy complications [21].

### Conclusion

We found lower rates of reproduction, and higher rates of sub-/infertility problems and pregnancy complications in VPT/VLBW females at the age of 35 years than in the general population. Personalized pre-conception counseling and close pregnancy monitoring may help VPT/VLBW females to beat the odds and complete their pregnancies with healthy outcomes for both mother and child.

## Data Availability

A request to obtain (aggregated) POPS-35 data can be submitted to pops@tno.nl or sylvia.vanderpal@tno.nl.

## Abbreviations

AGA: Appropriate for gestational age
ART: Assisted reproductive technology
BW: Birth weight
CBS: Dutch National Population Registry (CBS)
DOHAD: Developmental Origins of Health and Adult Disease
HDP: Hypertensive disorders of pregnancy
GA: Gestational age
PeriNed: Perinatal Registry in the Netherlands
POPS: Project on Preterm and Small for Gestational Age Infants
SGA: Small for gestational age
VPT: Very preterm
VLBW: Very low birth weight
